# Latent disease similarities and therapeutic repurposing possibilities uncovered by multi-modal generative topic modeling of human diseases

**DOI:** 10.1093/bioadv/vbad047

**Published:** 2023-04-12

**Authors:** Satoshi Kozawa, Hirona Yokoyama, Kyoji Urayama, Kengo Tejima, Hotaka Doi, Shunki Takagi, Thomas N Sato

**Affiliations:** Karydo TherapeutiX, Inc., Kyoto 619-0288, Japan; The Thomas N. Sato BioMEC-X Laboratories, Advanced Telecommunications Research Institute International (ATR), Kyoto 619-0288, Japan; ERATO Sato-Live Bio-Forecasting Project, Japan Science and Technology Agency (JST), Kyoto 619-0288, Japan; Karydo TherapeutiX, Inc., Kyoto 619-0288, Japan; The Thomas N. Sato BioMEC-X Laboratories, Advanced Telecommunications Research Institute International (ATR), Kyoto 619-0288, Japan; V-iCliniX Laboratory, Nara Medical University, Nara 634-8521, Japan; Karydo TherapeutiX, Inc., Kyoto 619-0288, Japan; The Thomas N. Sato BioMEC-X Laboratories, Advanced Telecommunications Research Institute International (ATR), Kyoto 619-0288, Japan; ERATO Sato-Live Bio-Forecasting Project, Japan Science and Technology Agency (JST), Kyoto 619-0288, Japan; Karydo TherapeutiX, Inc., Kyoto 619-0288, Japan; The Thomas N. Sato BioMEC-X Laboratories, Advanced Telecommunications Research Institute International (ATR), Kyoto 619-0288, Japan; ERATO Sato-Live Bio-Forecasting Project, Japan Science and Technology Agency (JST), Kyoto 619-0288, Japan; Karydo TherapeutiX, Inc., Kyoto 619-0288, Japan; The Thomas N. Sato BioMEC-X Laboratories, Advanced Telecommunications Research Institute International (ATR), Kyoto 619-0288, Japan; V-iCliniX Laboratory, Nara Medical University, Nara 634-8521, Japan; Karydo TherapeutiX, Inc., Kyoto 619-0288, Japan; The Thomas N. Sato BioMEC-X Laboratories, Advanced Telecommunications Research Institute International (ATR), Kyoto 619-0288, Japan; Karydo TherapeutiX, Inc., Kyoto 619-0288, Japan; The Thomas N. Sato BioMEC-X Laboratories, Advanced Telecommunications Research Institute International (ATR), Kyoto 619-0288, Japan; ERATO Sato-Live Bio-Forecasting Project, Japan Science and Technology Agency (JST), Kyoto 619-0288, Japan; V-iCliniX Laboratory, Nara Medical University, Nara 634-8521, Japan

## Abstract

**Motivation:**

Human diseases are characterized by multiple features such as their pathophysiological, molecular and genetic changes. The rapid expansion of such multi-modal disease-omics space provides an opportunity to re-classify diverse human diseases and to uncover their latent molecular similarities, which could be exploited to repurpose a therapeutic-target for one disease to another.

**Results:**

Herein, we probe this underexplored space by soft-clustering 6955 human diseases by multi-modal generative topic modeling. Focusing on chronic kidney disease and myocardial infarction, two most life-threatening diseases, unveiled are their previously underrecognized molecular similarities to neoplasia and mental/neurological-disorders, and 69 repurposable therapeutic-targets for these diseases. Using an edit-distance-based pathway-classifier, we also find molecular pathways by which these targets could elicit their clinical effects. Importantly, for the 17 targets, the evidence for their therapeutic usefulness is retrospectively found in the pre-clinical and clinical space, illustrating the effectiveness of the method, and suggesting its broader applications across diverse human diseases.

**Availability and implementation:**

The code reported in this article is available at: https://github.com/skozawa170301ktx/MultiModalDiseaseModeling

**Supplementary information:**

Supplementary data are available at *Bioinformatics Advances* online.

## 1 Introduction

Human diseases are characterized by alterations in a multitude of features: genetics, molecular, cellular, inter-organ pathways, histopathology, physiology, microbiota, etc. Recently, this disease-omics data space is rapidly expanding and becoming readily available, enabling the comprehensive characterizations of diverse human diseases (Hasin *et al.*, 2017; Perakakis *et al.*, 2018; Reel *et al.*, 2021).

For example, GWAS catalog (https://www.ebi.ac.uk/gwas/) and other similar databases provide a comprehensive list of genetic factors associated with thousands of human diseases and traits. KEGG (https://www.genome.jp/kegg/), Reactome (https://reactome.org) and other similar databases describe comprehensive molecular pathways. DisGeNET (https://www.disgenet.org) compiles altered gene expression, biomarkers, post-translational modifications, genetic factors, drug-targets, etc. and their association with human diseases. Disbiome (https://disbiome.ugent.be/home) tabulates human disease-associated microbiota. There are also large numbers of drug-related databases (SIDER: http://sideeffects.embl.de, DrugBank: https://go.drugbank.com, FAERS: https://www.fda.gov/drugs/questions-and-answers-fdas-adverse-event-reporting-system-faers/fda-adverse-event-reporting-system-faers-public-dashboard, etc.) that comprehensively list therapeutic-indications, side-effects/adverse-events, targets, etc. of the drugs. Moreover, we can virtually identify cells and organs that express genes/proteins of interest at The Human Protein Atlas (https://www.proteinatlas.org), Human Cell Atlas (https://www.humancellatlas.org) and other similar open-resources.

Hence, this rapidly expanding multi-modal disease-omics data space provides an opportunity to re-classify diverse human diseases according to their multi-modal similarity metrics. Furthermore, this approach could find previously underrecognized disease–disease similarities.

The pioneering study built on the graph theory provided an overview of disease–disease similarities according to their single modality features, genetic variabilities (Goh *et al.*, 2007). Since then, more sophisticated network-based and other approaches have evolved to characterize multi-modal nature of human diseases (Barabási *et al.*, 2011; García Del Valle *et al.*, 2021; Li *et al.*, 2021; Menche *et al.*, 2015; Perakakis *et al.*, 2018; Reel *et al.*, 2021). Despite the development of such tools and methods, the ever-expanding multi-modal disease-omics space remains under-explored. Hence, further in-depth probing of this data space is expected to uncover latent molecular mechanisms underlying non-classical under-recognized disease–disease similarities.

As an approach that could integrate multiple types of features to classify human diseases and measure their similarity metrics, topic modeling was brought to our attention. This algorithm has been applied to categorize social media information (Zheng *et al.*, 2014), and also to image annotation and classification and computer vision (Roller and im Walde, 2013). This approach has also recently been applied to the classification of clinical notes (Wen *et al.*, 2021) and RNA dual-omics (RNA, microRNA) data (Valle *et al.*, 2022).

Based on these previous reports, we considered a use of the multi-modal topic modeling to re-classify diverse types of human diseases according to their multi-modal disease-omics features. Using this multi-modal omics-based soft-clustering of human diseases, we aim to identify molecular targets that could be repurposed from one disease to another for its treatment and/or detection (i.e. latent disease-omics features/therapeutic-targets). Furthermore, the identification of unexpected repurposable therapeutic targets may unveil previously underrecognized disease–disease similarities (i.e. latent disease similarities). This approach differs from that of ‘drug-repurposing’ where a drug, not a disease omics-feature, for a disease is repurposed to treat another disease according to the similarities of drug characteristics (e.g. the similarities of drug structures, drug-targets, etc.) (Al-Saleem *et al.*, 2021; Bisgin *et al.*, 2012; Hooshmand *et al.*, 2021; Malas *et al.*, 2019; Panchapakesan and Pollock, 2018; Park, 2019; Pushpakom *et al.*, 2019; Schuler *et al.*, 2022).

Hence, in this article, we report a multi-modal generative topic modeling-based method that is suitable for re-classifying human diseases to identify repurposable molecular therapeutic-targets and to unveil latent disease–disease similarities. We also illustrate its applications to two globally most life-threatening human diseases, chronic kidney diseases (CKD) (Chen *et al.*, 2019) and myocardial infarction (MI) (Anderson and Morrow, 2017).

## 2 Methods

### 2.1 General overview of the multi-modal generative topic modeling approach in this study

**Fig. 1.**
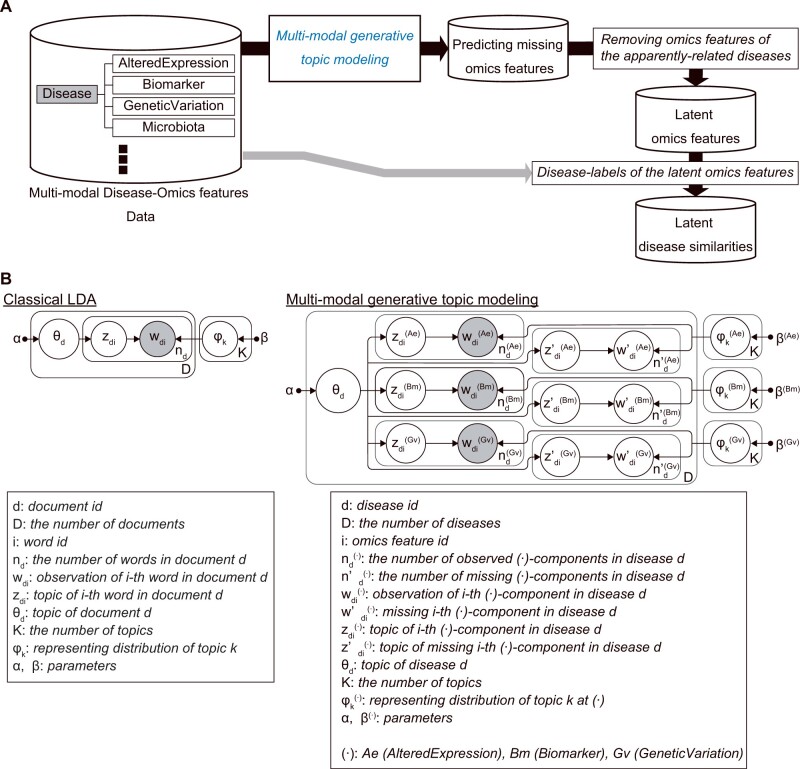
Schematic diagram of the multi-modal generative topic-modeling. (**A**) The general overview of the approach. Using the comprehensive multi-modal disease-omics datasets, the human diseases are soft-clustered by the multi-modal generative topic modeling according to their multi-modal similarity metrics. Next, the features of a modality of a disease-of-interest are intentionally removed (indicated as ‘missing omics features’ in the figure). These missing features are then predicted by the multi-modal generative topic model. From these predicted features, those of the apparently related diseases (e.g. myocardial infarction versus heart failure, chronic kidney disease versus renal failure, etc.) in the original datasets are then removed, leaving the unexpected features (‘latent omics features’) of the target diseases. As the result, the origin of the disease(s) of the latent omics features in the databases represents underrecognized latent disease–disease similarity. The datasets and their uses/analyses are illustrated as ‘cylinders’ and ‘boxes’, respectively. (**B**) The side-by-side comparison between the classical LDA algorithm and the multi-modal generative topic modeling introduced in this article. See also Sections 2.1, 2.2 and 2.3 for the details

The general overview of the approach in this study is shown in Figure 1A. The details are described in the following sections (Sections 2.2 and 2.3). Let us explain the overall approach by taking an example of predicting latent omics features and disease-similarities of CKD. The training dataset consists of 6955 human diseases, each of which is described by three disease omics modalities, AlteredExpression (Ae), Biomarker (Bm) and GeneticVariation (Gv) (see Section 2.2 for the details). Each modality contains distinct types of omics features that characterize the corresponding disease. First, we remove Ae modality for CKD, leaving the CKD training dataset with only two modalities, Bm and Gv. All the other diseases (6954 diseases) remain labeled with three modalities. By the multi-modal generative topic modeling (see Section 2.3 for the details), we predict the omics features of the missing Ae modality for CKD. We repeat this step for all three modalities (Ae, Bm, Gv) for CKD. Next, from the predicted omics features for all three modalities for CKD, we remove those included in the modalities of the diseases that are apparently related to CKD (e.g. renal diseases, coronary diseases, diabetes, etc.) (see Section 2.3.5 for the specific list of the diseases). Consequently, the remaining features represent the ‘latent omics features’ of CKD. We then identify the disease-labels of these latent omics features in the training dataset, and they constitute the list of the diseases of which similarity to CKD is ‘latent’ (i.e. underrecognized). Hence, this relationship is referred to as ‘latent disease similarity’ for CKD.

### 2.2 Multi-modal disease-omics datasets

The multi-modal disease-omics features for human diseases used in this study are AlteredExpression (Ae), Biomarker (Bm), GeneticVariation (Gv) data from DisGeNET v7.0. (https://www.disgenet.org/downloads) (Piñero *et al.*, 2017) and Microbiota (Mb) from Disbiome (version on 11th of November 2020, https://disbiome.ugent.be/home) (Janssens *et al.*, 2018). Ae is the list of genes and proteins of which changes in expressions are attributed to a disease(s). Bm is the list of biomarkers which are attributed to a disease(s). Gv is the list of genes of which mutations are reported for a disease(s). Mb is the list of microbial organisms of which changes in abundance are reported for a disease(s). We chose these molecular omics features to characterize and model human diseases as they are amenable to therapeutic-targeting and/or disease-detection. A total of 6955 and 158 human diseases are found labeled by all the combinations of the Ae/Bm/Gv and the Ae/Bm/Gv/Mb modalities, respectively.

To match disease names acquired from multiple data sources, we added UMLS IDs to the disease names. The UMLS ID annotation was performed by ‘UMLS_AUI.extract_terminology (“ICD10”)’ function from Python library ‘PyMedTermino (version 0.3.3)’ (Lamy *et al.*, 2015). Prior to performing this function, ‘’s’ was replaced by a blank space in disease names. Following the assignment of the UMLS ID annotations, the UMLS IDs were combined by string ‘|’ if these UMLS IDs have the same disease names. For the disease names where this UMLS ID annotation method failed, the actual disease names in the datasets were used and only those with the exact matching names were combined. For the Ae/Bm/Gv and the Ae/Bm/Gv/Mb combinations, a total of 1809 and 116 diseases are convertible to the UMLS IDs, respectively.

### 2.3 Multi-modal generative topic modeling of human diseases

#### 2.3.1 The multi-modal generative topic modeling and prediction of latent omics features

The multi-modal generative topic modeling that we employed is based on Latent Dirichlet Allocation (LDA) (Blei *et al.*, 2003). The side-by-side comparison between the classical LDA and our multi-modal generative topic modeling is shown in Figure 1B.

The classical LDA is commonly used to soft-clustering documents by using words in the documents (referred to as ‘bag-of-words’). Our multi-modal generative topic modeling is developed to soft-cluster human diseases based on their multi-modal disease-omics features (indicated as ‘components’ in the algorithm diagram) (e.g. Ae, Bm, Gv, Mb, etc.). In our multi-modal generative topic modeling, the human diseases and omics-features correspond to ‘documents’ and ‘words’ in the classical LDA model, respectively. Therefore, the human diseases are soft-clustered according to the co-occurrence of omics-features between the diseases. Furthermore, the model is generated by ‘cross-referencing’ the probabilistic distributions of the features of each other’s modality datasets, not by a simple concatenation of the multi-modal datasets. This is necessary as the omics features across the different modalities represent distinct types of the data—for example, Ae and Mb consist of the transcripts (i.e. mRNA) and microbial organisms, respectively. Therefore, the concatenation of such multi-modal data leads to inaccurate representation of human diseases.

In our multi-modal generative topic modeling, all modalities (i.e. Ae, Bm, Gv, Mb in this study) for a given disease are designed to exhibit the same probabilistic distribution pattern across the topics. Thus, the human diseases are soft-clustered according to the overall probabilistic distributions of the multiple modalities.

The probabilistic generative topic model using multi-modal datasets is built as follows (see also the right panel in Figure 1B for the graphical description): Let $w_{di}^{\left(\cdot \right)}$ be the i-th disease-omics component [we use ‘component(s)’ in the algorithm/program, but it is the same as the disease-omics feature(s)] of disease d acquired from the modality (i.e. Ae, Bm, Gv or Mb) dataset (⋅). Let $z_{di}^{\left(\cdot \right)}$ be the topic number of $w_{di}^{\left(\cdot \right)}$, $\theta_{d}$ be the topic probability of disease d, and $\phi_{k}^{\left(\cdot \right)}$ be the occurrence probability of disease-omics component v of topic k of (⋅) dataset. The topics number K is determined as described in Section 2.3.3. The joint distribution of these variables is defined as follows


(1) pw, z, θ, ϕα, β=∏⋅∏d∏ipwdi⋅zdi⋅,ϕ⋅∏⋅∏d∏ipzdi⋅θd× ∏dpθdα∏⋅∏kpϕk⋅β⋅.

The exact formula of the elements of the joint distribution is described as: where $\alpha \in R^{+K}$ and $\beta^{\left(\cdot \right)}\in R^{+V^{\left(\cdot \right)}}$ are hyper parameters which are set to vectors having the elements of 0.1, $V^{\left(\cdot \right)}$ is the total number of unique components across all diseases for the corresponding (⋅) dataset, Γ(⋅) is the Gamma function and δ(⋅) is the Kronecker delta function.


(2) pwdi⋅zdi⋅, ϕ⋅ =Multinomialwdi⋅ϕ⋅ =∏k=1K ∏v=1V⋅ϕkv⋅δzdi⋅=kδwdi⋅=v,

(3) pzdi⋅θd=Multinomialzdi⋅θd=∏k=1Kθdkδzdi⋅=k, 

(4) pθdα=Dirichletθdα=Γ∑kαk∏kΓαk ∏k=1Kθdkαk-1,

(5) pϕk⋅β⋅=Dirichletϕk⋅β⋅=Γ∑vβv⋅∏vV⋅Γβv⋅ ∏v=1V⋅ϕ⋅kvβv⋅-1,

#### 2.3.2 Estimate of the topic distribution and disease-omics components

Based on the model, we estimate the posterior distributions of the variables $z_{di}^{\left(\cdot \right)}$, $\theta_{d}$ and $\phi_{k}^{\left(\cdot \right)}$, and also estimate a part of $w_{di}^{\left(\cdot \right)}$ (it will be referred to as $w_{di}^{\prime \left(\cdot \right)}$ that is missing in a (⋅) dataset) by using Gibbs sampling method. Based on the generative model built as described in 2.3.1, the conditional distributions of the variable are calculated as follows:


(6) pzdi⋅=kwdi⋅=v,θd,ϕ⋅=θdkϕkv⋅∑k'θdk'ϕk'v⋅,

(7) pθdz⋅,α=Dirichletθdα'∝∏k=1Kθdkαk+∑iδzdi⋅=k-1,

(8) pϕk⋅z⋅, w⋅, β⋅=Dirichletϕk⋅β⋅'∝∏vϕ⋅kvβk⋅+∑d∑iδzdi⋅=k δwdi⋅=v-1,

(9) pwdi⋅=vzdi⋅=k, ϕ⋅=ϕkv⋅.

From these conditional distributions, we sample $z_{di}^{\left(\cdot \right)}$,$\theta_{d}$, $\phi_{k}^{\left(\cdot \right)}$ and ${w^{\prime }}_{di}^{\left(\cdot \right)}$, until their values converge. We found the topic distribution converges after 1000 to 2000 iterations with any combinations of the diseases and modalities, indicating that at least 2000 iterations are required. Therefore, to be sufficient enough, the number of iterations is set to 5000 in this article. After the completion of sampling, we estimate the value of each variable by averaging the sampled values from the conditional distributions. The initial values of the variables are set as follows: The initial values of $\theta_{d}$ and $\phi_{k}^{\left(\cdot \right)}$ were set to the uniform distribution. The initial value of ${w^{\prime }}_{di}^{\left(\cdot \right)}$ is set to the disease-omics component that is sampled from $\phi_{k}^{\left(\cdot \right)}$ at random. The total number of ${w^{\prime }}_{di}^{\left(\cdot \right)}$ of disease d is decided by sampling from the binominal distribution. The parameters for the binominal distribution are estimated by the maximum likelihood estimation method using the observed data. The estimated value of $\theta_{d}$ represents the probability of topics at disease d. The likeliness of the missing values of disease d in (⋅) dataset can be inferred by sampling frequency of ${w^{\prime }}_{di}^{\left(\cdot \right)}$.

#### 2.3.3 Computation of the topics number K

The topics number K is selected by the Louvain method (Blondel *et al.*, 2008). To apply this method, an undirected graph per each of the Ae, Bm, Gv, Mb datasets is constructed by using ‘NetworkX’ (https://networkx.org/). In each graph, if two diseases (i.e. nodes) share disease-omics components, we allow an edge between them. The edges are weighted according to the number of shared disease-omics components. The Louvain method is then applied 20 times to each graph (i.e. Ae, Bm, Gv, Mb modality), and the most frequently obtained number of communities was selected for the community number for each modality. The maximum community number for the combination of the modalities (Ae/Bm/Gv or Ae/Bm/Gv/Mb) is then selected as the K for the corresponding modality combination. The computed topics numbers are 6 and 3 for the Ae/Bm/Gv and Ae/Bm/Gv/Mb combinations, respectively (Supplementary Fig. S1). The Louvain method is performed by Python package ‘python-louvain’ (https://python-louvain.readthedocs.io/en/latest/).

#### 2.3.4 Performance evaluation of the multi-modal generative topic modeling method

The performance of the multi-modal generative topic modeling is evaluated by ‘leave-one-modality-out’ per each disease method and then by calculating their AUC scores. The input is the Ae/Bm/Gv or Ae/Bm/Gv/Mb dataset where single modality components (e.g. Ae, Bm, Gv, Mb) are purposely left-out for each disease. We then performed the multi-modal generative topic modeling as described in the previous sections on each of these input datasets. The likeliness of the missing disease-omics features is determined by the sampling frequency of ${w^{\prime }}_{di}^{\left(\cdot \right)}$, where (⋅) is either Ae/Bm/Gv or Ae/Bm/Gv/Mb combination. Hence, the likeliness is the prediction probability of the missing disease-omics component ${w^{\prime }}_{di}^{\left(\cdot \right)}$. The label is defined whether each of the disease-omics components is present or not (i.e. binary labeling) in the original dataset. The AUC scores are calculated from these prediction probability values and the labels for the disease in each of the (⋅) datasets. The calculation of the AUC scores was performed by function ‘roc_auc_score()’ in package ‘scikit-learn’ (https://scikit-learn.org/stable/) (Pedregosa *et al.*, 2011). The Youden’s index (Youden, 1950) was used as the cut-off threshold for the sampling frequency of ${w^{\prime }}_{di}^{\left(\cdot \right)}$. Youden’s index is a ROC curve-based thresholding method. The thresholds for each modality for each disease were computed as follows: (i) true positive rate (TPR) and false positive rate (FPR) were computed using the function ‘roc_curve()’ in the package ‘scikit-learn’ (https://scikit-learn.org/stable/), (ii) the Youden’s index was then calculated by the formula, TPR-FPR for each threshold and (iii) for each modality of each disease, the threshold which corresponds to the maximum Youden’s index was selected as the cut-off threshold for the corresponding modality for each disease.

#### 2.3.5 Identification of latent disease-omics features and disease-similarity

The latent disease-omics features are identified by removing the features derived from the diseases (in the training dataset) that are apparently related to the target disease(s). For CKD and/or MI as the target diseases, the removed are the features derived from the diseases of which names contain the following terms: ‘heart’, ‘**cardi**’(e.g. myocardial), ‘athero**’ (e.g. atherosclerosis), ‘arterio**’ (e.g. arteriosclerosis), ‘coronary’, ‘kidney’, ‘renal’, ‘nephro**’ (e.g. nephropathy), ‘glomer**’ (e.g. glomerular), ‘diabe**’ (e.g. diabetes), ‘vascul**’ (e.g. vascular), ‘capil**’ (e.g. capillary), ‘hypertens**’ (e.g. hypertension) (** could be any characters).

Next, the latent disease-similarity is determined as follows: The disease-labels of these latent disease omics features in the training dataset are identified. Hence, they represent the diseases of which similarity to the target diseases (i.e. CKD and/or MI in this study) is latent. Therefore, this disease–disease relationship is referred to as ‘latent disease-similarity’.

### 2.4 Characterization of the latent disease-omics features

#### 2.4.1 Organ/cell expression enrichment analysis

To find the specific organ/cell-expression patterns for the identified latent disease-omics features, we performed human organ/cell enrichment analyses using THE HUMAN PROTEIN ATLAS v 21.1. (https://www.proteinatlas.org) (Thul *et al.*, 2017; Uhlén *et al.*, 2015). The table was downloaded from ‘25. Data from the Human Protein Atlas in tab-separated format’ in the ‘DOWNLOADABLE DATA’ page (https://www.proteinatlas.org/about/download). The enrichment analysis was performed using chi-square test of independence to evaluate the statistical significance of the enriched expression in the specific organ(s)/cell(s) detected for the genes of interest. We performed the test by making the 2 × 2 contingency table consisting of the appearance frequency of the genes of interest and that of the genes of interest in each organ/cell. This table was used as the input to perform the chi-square test of independence using the Python function ‘scipy.stats.chi2_contingency()’ (https://docs.scipy.org/doc/scipy/index.html).

#### 2.4.2 KEGG enrichment analysis

To find the specific KEGG pathways for the predicted latent disease-omics features, we performed KEGG enrichment analysis using KEGG database (https://www.genome.jp/kegg/) (Kanehisa and Goto, 2000). KEGG enrichment analysis was performed by R function ‘enrichKEGG()’ in the package ‘clusterProfiler’ (Yu *et al.*, 2012). For inputting enrichKEGG(), the symbol names of the genes were converted to Entrez IDs using R function ‘bitr()’ in the package ‘clusterProfiler’ (Yu *et al.*, 2012).

#### 2.4.3 GO enrichment analysis

To find the specific gene ontology terms for the predicted latent disease-omics features, we performed GO enrichment analysis using GO database (http://geneontology.org/) (Ashburner *et al.*, 2000; Carbon *et al.*, 2021). GO enrichment analysis was performed by R function ‘enrichGO()’ in package ‘clusterProfiler’ (Yu *et al.*, 2012). For inputting enrichGO(), the symbol names of the genes were converted to Entrez IDs using R function ‘bitr()’ in the package ‘clusterProfiler’ (Yu *et al.*, 2012).

### 2.5 Edit-distance-based classifier

Edit-distance-based method was designed for two purposes:

1. To measure the relatedness of the sequential orders of the molecular components in the molecular pathways triggered by the predicted latent omics-features/components to the known molecular pathways for their target disease (CKD or MI in this article).
2. To infer putative side-effects that could result from targeting the predicted latent omics-features/components.

The overall design of the method, consisting of multiple modules, is schematically shown in Figure 7. We employed two methods, Method A and Method B. Each module is as described below:

#### 2.5.1 Path extraction

Each path is a sequence of KEGG components extracted from the KEGG pathway. In the KEGG database, all components are systematically labeled as identifiers (e.g. hsa:3065). Therefore, we use the identifiers, allowing the reliable computation of the distances. Each path starts at the node of a KEGG component described as a drug target at the DrugBank (https://go.drugbank.com/) and ends at the node of a KEGG component with an outdegree of 0. The therapeutic indications (TIs) and side-effects (SEs) for each drug for the drug target(s) are from SIDER 4.1 (http://sideeffects.embl.de/). This design results in the paths where the starting nodes (i.e. molecular targets of the drugs) are labeled with TIs and SEs. The holdout validation was performed with the training versus test data as 9:1.

#### 2.5.2 Similarity computation by edit-distance

The similarity between two paths N and M is calculated using the edit-distance (Levenshtein distance) method as follows: where length(X) is the number of components of path *X*. The edit-distance between paths *N* and *M* is calculated by considering a path as a word and a component as a character, using the dynamic programming algorithm (Navarro, 2001) as follows: where $N_{i}$ is the ith component of path N and $M_{j}$ is the jth component of path M.


(10) SimilarityN,M=1-EditDistanceN,Mmax⁡lengthN, lengthM,

(11) Ci,0=i 0≤i≤lengthN,C0,j=j0≤j≤lengthM,

(12) x=0 if Ni=Mj1 if Ni≠Mj,

(13) Ci,j=min⁡Ci-1,j+1, Ci,j-1+1, Ci-1,j-1+x,

(14) EditDistanceN,M=ClengthN,lengthM,

#### 2.5.3 Feature selection of the paths for TI/SE by PCA (Method A)

For the target TI or SE, we calculated the similarity matrix A between the paths with a starting node for the selected TI or SE and the paths for the training or prediction (Fig. 7A). Each component of matrix A is the pairwise similarity value calculated by the edit-distance as described in Section 2.5.2. The dimension of matrix A is reduced by the principal component analysis (PCA) (Wold *et al.*, 1987). The principal components are selected until the cumulative contribution reaches 99%. The PCA result was used as the features of the paths for the training or prediction for the target TIs or SEs.

#### 2.5.4 Feature selection of the paths for TI/SE by clustering (Method B)

For each drug target *x*, we calculated the similarity matrix *B* between the paths with *x* as a starting node and all paths (including the paths with *x* themselves) (Fig. 7B). Each component of matrix *B* is the pairwise similarity value calculated by the edit-distance as described in Section 2.5.2. The correlation matrix *C* of the paths with a starting node x was calculated by considering the row of matrix *B* as the vector of the path. A clustering for these paths was performed by the Python function ‘scipy.cluster.hierarchy.average()’ (https://scipy.org/) using matrix *C*. The clusters are composed of paths whose distance from each other is less than or equal to the threshold of 0.2. Each of the clusters (e.g. Cluster 0, Cluster 1, etc.) is then linked to each path. Next, for each path with a starting node corresponding to the selected target TI or SE, its starting node is paired with its cluster number (Fig. 7C). The sampling is performed as follows. If the multiplicity of the pair(s) is greater than ceil(500/*n*), where n is the number of the pairs excluding duplicates, the corresponding paths were sampled to ceil(500/*n*). If the multiplicity of the pair(s) is less than or equal to ceil(500/*n*), the corresponding paths were used as they are. The ceil(500/*n*) was selected as the maximum value within our hardware capacity. The similarity matrix between the paths obtained by this method and paths for the training or prediction was calculated and used as the feature of the selected TI or SE. Each component of the similarity matrix is the pairwise similarity value calculated by the edit-distance as described in Section 2.5.2.

#### 2.5.5 Training of binary classifier

For the target TI or SE, the paths for the training were downsampled to the ratio of 1(positive):1(negative) (the paths for the corresponding TI or SE versus the paths that do not correspond to the target TI or SE). Random under-sampling was performed on Python package ‘imblearn’ (https://imbalanced-learn.org/stable/index.html) (Lemaitre *et al.*, 2017). These downsampled paths were converted to features by using ‘Method A’ or ‘Method B’ as described in 2.5.3 or 2.5.4, respectively. Ten models determining whether or not given paths are related to each of the TIs or SEs were developed for bagging using the downsampled path-TI (or SE) features and trained by LightGBM (Ke *et al.*, 2017). This training process was conducted for all TIs and SEs. The hyperparameters of LightGBM were tuned by Python function ‘optuna.integration.lightgbm()’ (https://optuna.org/) using 20% of the downsampled data.

#### 2.5.6 Prediction of TIs and SEs

The paths with a drug-target component as the starting node were extracted from the KEGG pathways as described in the Section 2.5.1. The feature of paths was calculated by Methods A or B as described in Sections 2.5.3 or 2.5.4, respectively. These features were used as input data (Fig. 7D). For each TI or SE, if the predicted values for a given path show ≥0.5 with 6 or more of the 10 models, the path was determined as ‘true (1)’, and if the predicted values show otherwise, it was determined as ‘false (0)’.

### 2.6 The retrospective validation in the pre-clinical and clinical data space

To further evaluate the likeliness of the repurposability of the predicted target molecules for CKD and/or MI biomarkers and/or therapeutic-targets, we conducted comprehensive pre-clinical and clinical data-mining to retrospectively find any such implications in Google, PubMed, Cinicaltrials.gov (https://www.clinicaltrials.gov) data space.

## 3 Results

### 3.1 Multi-modal generative topic modeling of human diseases and its prediction performance

**Fig. 2.**
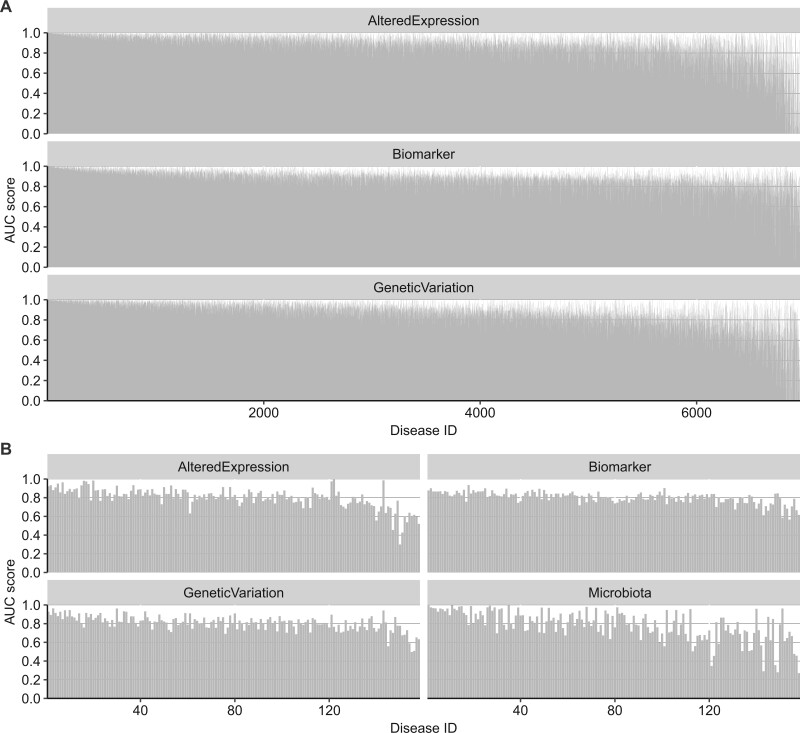
The AUC scores of the cross validations. (**A**) The AUC scores for the AlteredExpression, Biomarker, GeneticVariation modalities across 6955 human diseases. The raw data are available as Supplementary Table S1. (**B**) The AUC scores for the AlteredExpression, Biomarker, GeneticVariation, Microbiota modalities across 158 human diseases. The raw data are available as Supplementary Table S2

In this study, we developed a multi-modal generative topic modeling method that is applicable to multi-modal disease-omics data of human diseases (Fig. 1 and Sections 2.3.1, 2.3.2 and 2.3.3). We evaluated its prediction performance using Ae, Bm, Gv for 6955 human diseases derived from DisGeNET (https://www.disgenet.org). The prediction performance-validation is conducted by randomly dividing them into the 6665 diseases as training data and the remaining 290 diseases as test data encompassing all 6955 diseases. For each set of the test diseases, we removed their single modality features as prediction targets. We tested one modality at a time for each set of the test diseases. The prediction performance for the removed (i.e. missing in the test data) modality features in the test data is measured by Area Under receiver operating characteristic Curve (AUC) (Fig. 2A, Supplementary Table S1, see also Section 2.3.5). The results show that AUCs for approximately 88% (6125/6955), 92% (6381/6955), 84% (5812/6955) of the diseases are >0.8 for Ae, Bm, Gv, respectively, supporting the effectiveness of the method.

To test the modality-scalability of the method, we added another modality dataset, disease-microbiota (Mb) dataset from Disbiome (https://disbiome.ugent.be/home) to the above Ae/Bm/Gv combination. This expanded combination of datasets enabled us to soft-cluster 158 diseases all of which are annotated with the Ae, Bm, Gv and Mb features. The performance is evaluated by leaving-one-modality-of-a-disease-out validation and by computing the AUC for each modality (i.e. Ae, Bm, Gv, Mb) of each disease (Fig. 2B, Supplementary Table S2). The result shows that AUCs for approximately 59% (93/158), 58% (91/158), 61% (96/158), 50% (79/158) of the diseases are >0.8 for Ae, Bm, Gv, Mb, respectively. Despite the significant reduction of the training data (6955 diseases for the three-way modalities versus 158 diseases for the four-way modalities), more than 50% of the diseases exhibit the AUCs of > 0.8, supporting the reasonable modality-scalability of the method.

### 3.2 Inference of repurposable molecular targets for CKD and MI and their similarities to other diseases

**Fig. 3.**
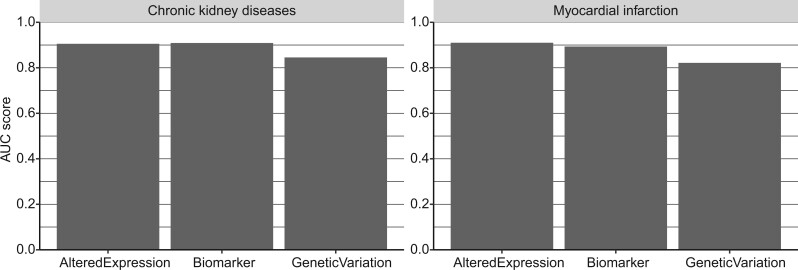
The AUC scores of each modality for the chronic kidney disease and the myocardial infarction. The AUC scores are calculated for AlteredExpression, Biomarker, GeneticVariation modalities for each disease (chronic kidney disease, myocardial infarction) and shown as bar graphs. The bars are shown as the AUC scores of 10× leave-one-modality/disease-out cross-validations. The raw data are available as Supplementary Tables S3–S18

Using this performance-validated method, we aimed to identify repurposable molecular targets for treating and/or detecting two globally most life-threatening diseases, CKD and MI. For this purpose, we further tested the performance of the method specifically for CKD and MI using the above-described Ae/Bm/Gv datasets encompassing 6955 diseases including CKD and MI. To infer repurposable Ae, Bm, Gv features from the other diseases as therapeutic-targets for CKD and/or MI, we purposely removed Ae, Bm or Gv features from CKD or MI and predicted the missing features. The result shows AUCs >0.8 for all Ae, Bm and Gv features of both diseases (Fig. 3, Supplementary Tables S3–S9).

**Fig. 4.**
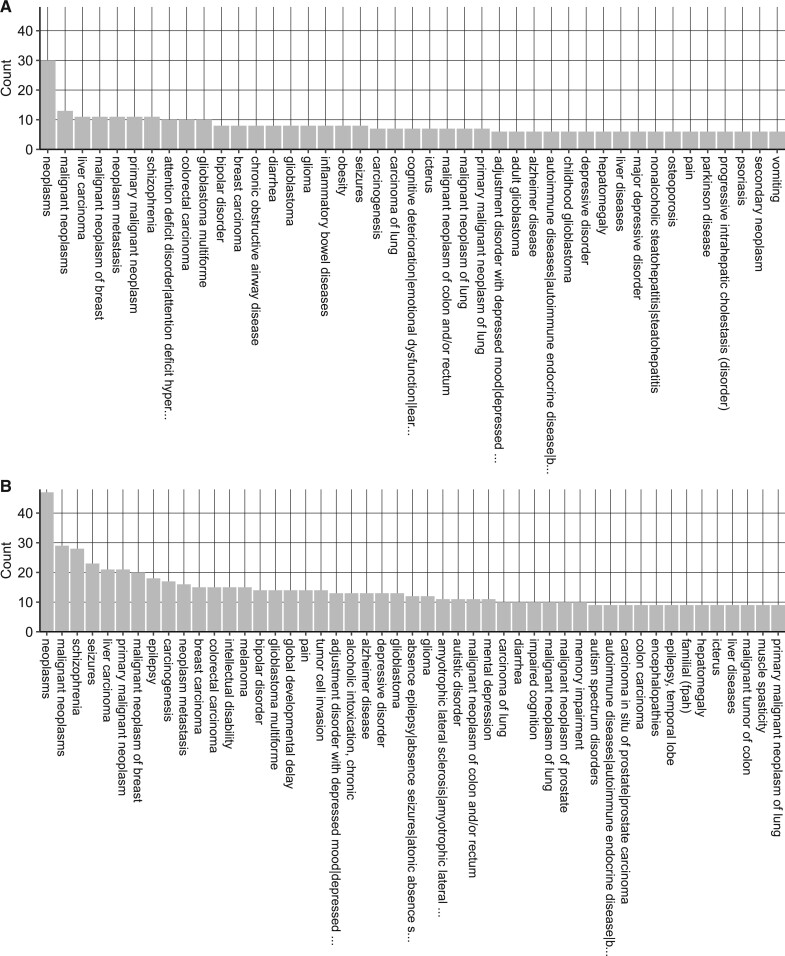
Enrichment analysis of the original human diseases in the DisGeNET database from which the latent disease-omics features for CKD and MI are identified. (**A**) CKD, (**B**) MI. The numbers of the predicted latent disease-omics features that appear in each disease are indicated as ‘count’. The long disease names are cut short and indicated as ‘.’ at their ends. The raw data are available as Supplementary Table S19

From this list, the features that are present in the training datasets (i.e. those correctly predicted) were first removed, leaving those that are absent in the CKD or MI data. Next, we used Youden’s index to select those that are considered as ‘statistically positive’ by this criterion (Supplementary Table S10, see also Section 2.3.4). Through these selections, left are features that are absent in the training datasets for the corresponding diseases and regarded as statistically significant (Supplementary Tables S11–S16). Further selection was conducted by removing those that are labeled with other kidney/renal and cardiac/heart/cardiovascular related diseases (e.g. renal failure, heart failure, etc.), as we could easily postulate, without any computational-methods, their repurposability to CKD and/or MI therapeutics. Through this additional selection step, we obtained a list of 30 and 57 molecular therapeutic candidates for CKD and MI, respectively, out of which 18 are shared by the two (Supplementary Tables S17 and S18). These candidates are particularly enriched in Ae, Bm and/or Gv of neoplasia (e.g. neoplasms, malignant neoplasms, neoplasm metastasis, malignant neoplasm of breast, primary malignant neoplasm, liver carcinoma, etc.) and mental/neurological disorders (e.g. schizophrenia, seizures, epilepsy, intellectual disability, etc.) (Fig. 4, Supplementary Table S19), unveiling their molecular similarities to the renal and/or cardiovascular diseases such as CKD and MI. Considering the relatively high AUCs of this inference method (Fig. 3), this possibility is further supported.

### 3.3 Therapeutic mechanisms of the inferred targets

**Fig. 5.**
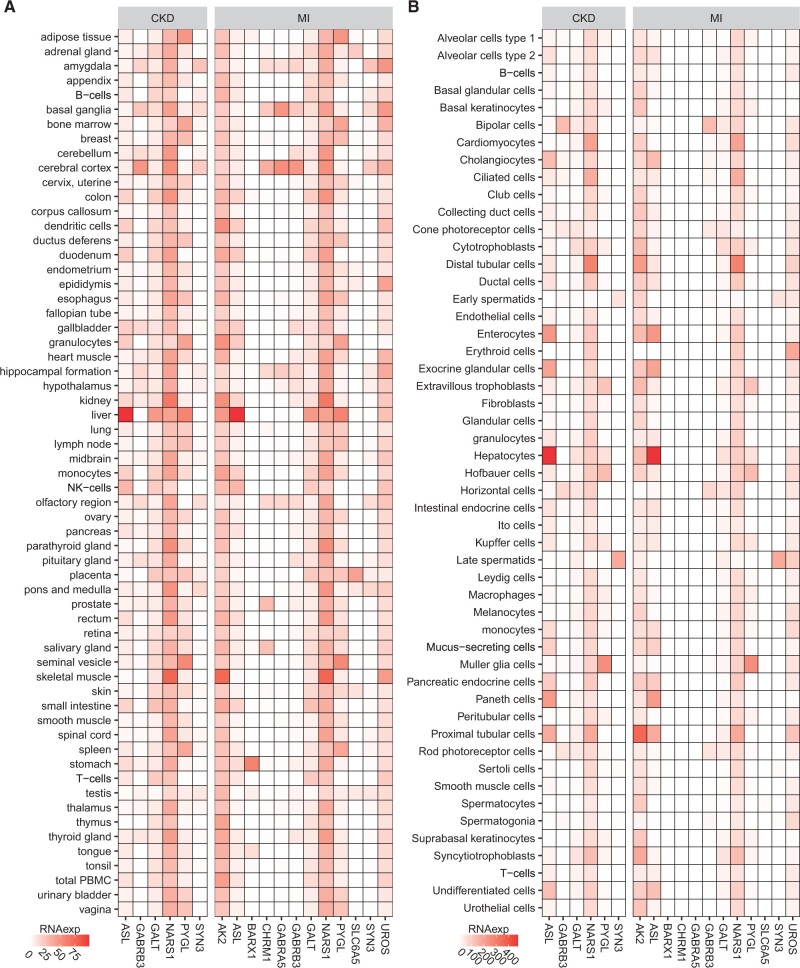

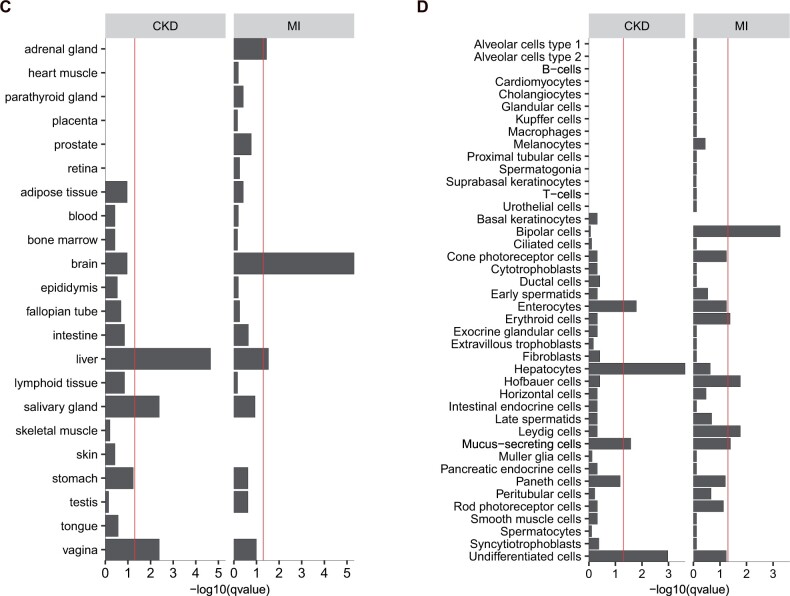
The organ/cell expression patterns of the predicted disease-omics features for CKD and MI. (**A**) The heatmap indicating the expression levels of each predicted target (bottom) in each organ (left) for each disease (top). The raw data are available as Supplementary Table S20. (**B**) The heatmap indicating the expression levels for each predicted target (bottom) in each cell-type (left) for each disease (top). The raw data are available as Supplementary Table S21. (**C**) The organ enrichment analysis result for the predicted targets for each disease (top). The enrichment levels are indicated as –log10(*q*-values). The *q*-value (*q*value)=0.05 is indicated as a red line in each graph. The raw data are available as Supplementary Table S22. (**D**) The cell-type enrichment analysis result for the predicted targets for each disease (top). The enrichment levels are indicated as –log10(*q*-values). The *q*-value (*q*value) = 0.05 is indicated as a red line in each graph. The raw data are available as Supplementary Table S23

To gain mechanistic insights into the putative therapeutic actions of the predicted target molecules, we identified their expression patterns in the human body using a comprehensive human protein/gene expression database (Fig. 5, see also Section 2.4.1). While the expression of each target is found across multiple organs and cell-types (Fig. 5A and B, Supplementary Tables S20 and S21), the CKD and MI targets are enriched in the liver and the brain, respectively (Fig. 5C, Supplementary Table S22). At the single cell-level, we observed some enrichment in the hepatocytes and bipolar cells for CKD and MI, respectively (Fig. 5D, Supplementary Table S23). The result suggests that these organs/cells may serve as therapeutic targets for the respective disease.

**Fig. 6.**
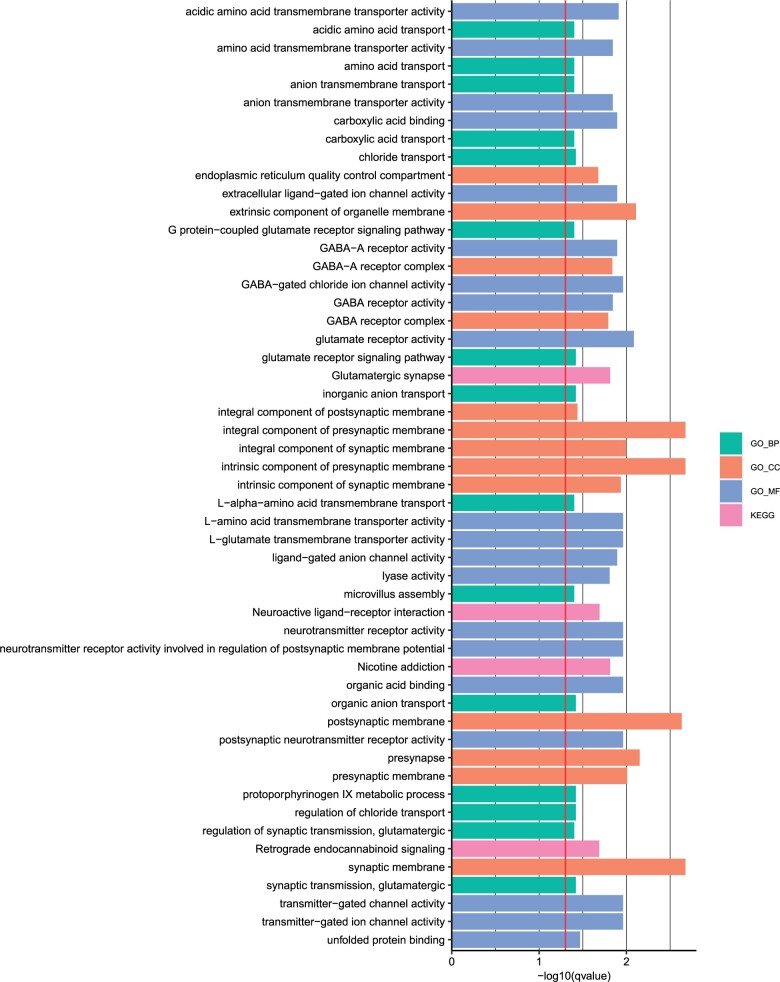
KEGG and GO enrichment analyses of the predicted disease-omics features for MI. The enrichment values for KEGG pathways and GO terms are shown as bars. The enrichment value is indicated as –log10(*q*-values). Those with *q*-values < 0.05 are shown. The *q*-value (qvalue)=0.05 is indicated as a red line in each graph. The raw data are available as Supplementary Tables S24–S28

Further mechanistic insights were gained by the enrichment analyses of biological pathways and functions using KEGG and GO databases (Fig. 6, Supplementary Tables S24–S28, see also Sections 2.4.2 and 2.4.3). The analyses found the enrichment of the MI targets in neural-pathways and -functions. These analyses, together with the expression pattern results, suggest that the nervous system functions and pathways are potential therapeutic targets for MI. In contrast, no enrichments are found for the CKD targets, instead they are sparsely encompassed across multiple biological pathways and functions (Supplementary Tables S25–S28).

**Fig. 7.**
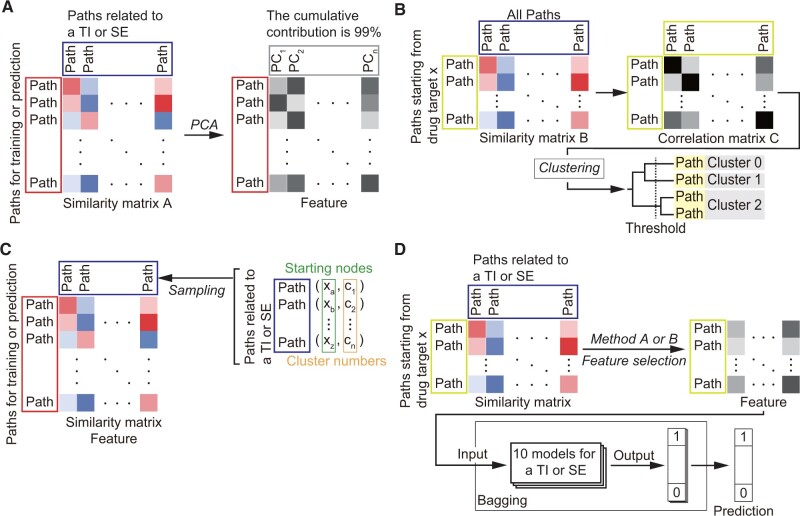
Schematic diagram of the edit-distance-based classifier. (**A**) The feature selection module for Method A. (**B**) The path—cluster linking module for Method B. (**C**) The feature selection module for Method B. (**D**) The prediction module for Methods A and B. See ‘Edit-distance-based classifier’ in the Section 2.5 for the detailed step-by-step description. The raw data are available as Supplementary Tables S29 and S30

Therapeutic-targeting elicits both favorable and unfavorable effects. The former is the therapeutic indications (TIs) and the latter is side-effects (SEs). Hence, we designed an analysis to infer these effects triggered by targeting the identified latent disease-omics features. For this purpose, we employed an edit-distance-based machine-learning classifier method (Fig. 7, see also Section 2.5). This classifier uses the edit-distance, specifically Levenshtein distance, to measure the similarity metrics between the pathways. This method measures the similarities of all possible pathways of the target candidates to each pathway downstream of the clinically approved drugs for each disease are computed. This is repeated for all pair-wise combinations for each disease and the computed edit-distances are used as input data for the corresponding disease-pathways classifier.

We, first, applied this method to the CKD and MI classifiers to determine the extent of the similarities between the pathways downstream of the identified latent disease-omics feature molecules and those of clinically approved drugs for CKD and/or MI. The hold-out validation shows that this method is highly reliable as indicated by the high prediction performance measures (i.e. accuracy scores > 0.94, precision scores > 0.71, recall scores > 0.85, F1 scores > 0.81) for both CKD and MI (Supplementary Tables S29 and S30).

**Fig. 8.**
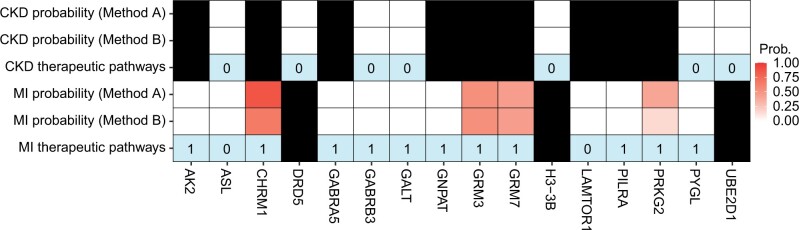
The deduced therapeutic pathways of the predicted targets. The probabilities that the identified latent omics-features elicit their predicted therapeutic effect(s) via the clinically approved therapeutic pathways for each target disease (CKD: chronic kidney disease, MI: myocardial infarction) are shown as heatmap (CKD/MI probability on the left). For which of the diseases (CKD versus MI) each target is predicted are indicated by open (predicted for) or filled (not-predicted for) cells. Whether the targets are within or outside the same KEGG pathway(s) of the known targets of the clinically approved drugs for the corresponding target disease are indicated as ‘1’ and ‘0’, respectively, in the corresponding cell (CKD/MI therapeutic pathways on the left). The raw data are available as Supplementary Tables S31–S34

Based on this highly reliable performance of the method, we applied it to infer molecular pathways impacted by the identified latent therapeutic targets (i.e. latent disease-omics features) (Fig. 8, Supplementary Tables S31–S34). This analysis found none of the CKD candidates share their pathways with the known CKD targets. Moreover, none belong to the same KEGG pathways. For MI, two candidates (ASL, LAMTOR1) are predicted as outside the known MI pathways, nor do they belong to the same KEGG pathways. Additionally, nine other candidates (AK2, GABRA5, GABRB3, GALT, GRM7, PILRA, PRKG2, PYGL, GNPAT) are also predicted as outside the known MI pathways, although they belong to the same KEGG pathways as the MI pathways. In contrast, two MI candidates (CHRM1, GRM3) and the known MI targets share the parts of their pathways.

**Fig. 9.**
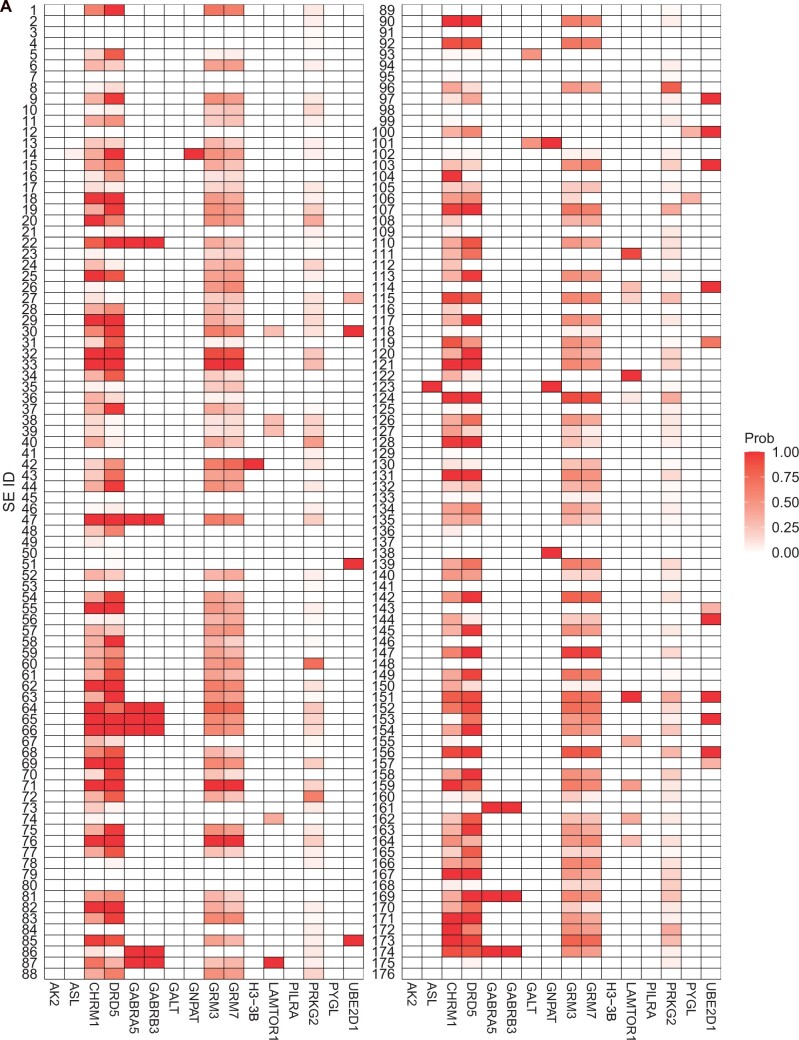

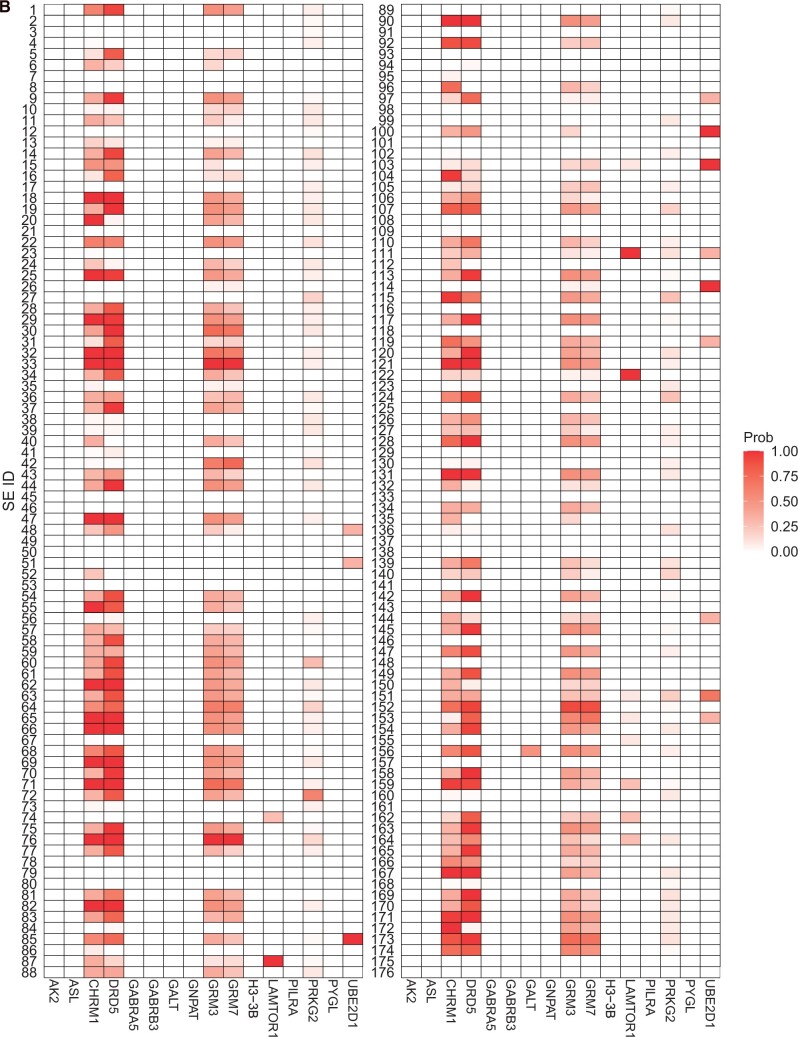
The side-effects inferred by the edit-distance-based classifier for the predicted disease omics-features upon their therapeutic-targeting. (**A**) The probabilities for the 176 serious side-effects (left) are indicated as the heatmap for the CKD targets (bottom). The raw data are available as Supplementary Table S35. (**B**) The probabilities for the 176 serious side-effects (left) are indicated as the heatmap for the MI targets (bottom). The raw data are available as Supplementary Table S35. Each side-effect is indicated as ID number (SE_ID) on the left of each panel and they are fully described in Supplementary Table S35

Next, we examined potential SEs resulting from targeting the identified latent disease-omics feature molecules (Fig. 9, Supplementary Table S35). In this study, we focused on the 176 serious adverse outcomes. The hold-out validation shows F1 scores > 0.5 for 124 out of the 176 SEs, suggesting that this prediction method is relatively useful. This prediction found four candidates (AK2, ASL, PILRA, PYGL) that are free of the selected 176 serious adverse outcomes, suggesting that they are less harmful therapeutic-targets.

### 3.4 Retrospective pre-clinical and clinical validation of the 69 targets

Next, we searched the publications and Clinicaltrials.gov (https://www.clinicaltrials.gov) for the predicted therapeutic utility of the 69 targets (Table 1, Supplementary Table S36, see also Section 2.6). This examination found pre-clinical and/or clinical evidence supporting the therapeutic usefulness of the 17 out of 69 targets for renal and/or cardiovascular diseases, providing the independent and retrospective validation of their predicted therapeutic repurposing possibilities.

**Table 1.** The retrospective validation of the predicted therapeutic targets in the pre-clinical and clinical data space

CKD	MI	Targets	Publication	ClinicalTrials.gov
	MI	ADRM1		
CKD	MI	AEBP1	DOI:10.1186/s12967-021-03000-3	
	MI	AK2	DOI:10.1016/j.bbrc.2021.01.097	
CKD	MI	ASL		NCT02252770
CKD	MI	ATP8B1		NCT02094222
	MI	BARX1		
CKD	MI	BLOC1S2	DOI:10.1038/cdd.2015.128	
	MI	C15orf41		
	MI	C19orf12		
	MI	C2-AS1		
	MI	CCDC115		
	MI	CD79B		NCT04668365
	MI	CHRM1		
CKD		CRNKL1		
CKD		DRD5	DOI:10.1681/ASN.2010050533	
	MI	ECT		
	MI	EDS8		NCT02165085
CKD	MI	EPB41		
	MI	ERLEC1		
CKD		ERVK-19		
CKD		FAM13A		
	MI	GABRA5		
CKD	MI	GABRB3		
CKD	MI	GALT		NCT02519504
	MI	GNPAT		
	MI	GRIK1		
	MI	GRM3		
	MI	GRM7		
CKD	MI	GUCY2C		NCT03217591
CKD		H3-3B	DOI:10.1038/s41598-018-32518-8	
	MI	HBB-LCR		
	MI	HBE1		
	MI	HM13		
CKD	MI	HSD3B7		
CKD		HTR3A		
	MI	IKZF2		
CKD		IL36RN	DOI:10.1016/j.kint.2017.09.017	
CKD	MI	IREB2		
	MI	LAMTOR1		
CKD		LINC01185		
	MI	LMF1		NCT03912181
CKD	MI	NARS1		
	MI	NDUFB2		
CKD		NME8		
	MI	ODAM		
	MI	P3H3		
	MI	PCDH19		
	MI	PILRA		
	MI	PRKG2		
CKD	MI	PYGL		NCT02385162
	MI	RANBP2		
	MI	RHAG		
	MI	SARM1		
	MI	SLC17A7		
CKD	MI	SLC25A13		
	MI	SLC6A5		
CKD	MI	SPAG8		
	MI	SPAST		
CKD	MI	SPATC1L	DOI:10.3892/mmr.2017.7334	
	MI	SPNS1		
	MI	STK26		
CKD	MI	SYN3		
CKD		THRSP	DOI:10.1155/2014/520281	
CKD	MI	TRP-AGG2-6		
CKD		UBE2D1	DOI:10.1155/2022/9463717	
CKD	MI	UGGT1		
	MI	UROS		
	MI	UST		
CKD		ZDHHC13		

*Note*: The publications and clinical-trials are indicated as DOI and clinicaltrial.gov NCT numbers, respectively. The blank cells are those without publications or clinicaltrials.gov evidence. The raw data are available as Supplementary Table S36.

These 17 targets are ATP8B1 (1/69), ASL (3/69), GUCY2C (4/69), GALT (8/69), BLOC1S2 (9/69), AEBP1 (10/69), CD79B (24/69), EDS8 (26/69), PYGL (29/69), IL36RN (30/69), LMF1 (35/69), THRSP (50/69), SPATC1L (53/69), H3-3B (54/69), DRD5 (56/69), UBE2D1 (57/69), AK2 (62/69), where the numbers in () are the ranking of each target among the 69 predicted targets (e.g. 1/69 indicating the 1st among the 69, 3/69 indicating the 3rd among the 69, etc.) according to their predicted probabilities (Supplementary Tables S4–S9).

The pre-clinical and clinical evidence include experimental results with knockout mice of the target gene(s) and the outcomes obtained from clinical-trials, respectively. For example, knockout mice for AK2, an MI candidate, are reported to show cardiac dysfunctions (Zhang *et al.*, 2021). This mouse study suggests a role of AK2 in cardiac development and/or function. Hence, AK2 could serve as a therapeutic target for cardiovascular diseases such as MI.

Deficiency of ASL, a candidate for both CKD and MI, is a rare genetic disorder resulting in argininosuccinic aciduria, a defective urea cycle condition leading to the insufficient breakdown/removal of nitrogen from the body, and consequently the patients develop hypertension (Kho *et al.*, 2018). Hypertension is a known risk factor for both renal and cardiovascular diseases including CKD and MI (Clemmer *et al.*, 2022; Ku *et al.*, 2019). A clinical trial (NCT02252770) was conducted to evaluate the benefit of a nitric oxide dietary supplementation to argininosuccinic aciduria patients, but no outcomes are yet reported.

A cross-transplantation study using the kidneys from the knockout mice of DRD5, a CKD candidate, shows hypertension and cardiac dysfunctions in this mouse model (Asico *et al.*, 2011). Furthermore, both hypertension and cardiac dysfunctions are known risk factors for renal diseases such as CKD (Clemmer *et al.*, 2022; Ku *et al.*, 2019), hence, supporting the prediction of DRD5 as a therapeutic candidate for CKD.

GUCY2C, guanylate cyclase 2C, is predicted as a therapeutic target for both CKD and MI (Table 1). In the clinical trial (NCT03217591), therapeutic effects of a soluble guanylate cyclase stimulator, IW-1973 (a.k.a., Praliciguat) for diabetic nephropathy/diabetic kidney diseases were evaluated. The outcomes show several metrics supporting further investigation of Praliciguat for diabetic kidney diseases (Hanrahan *et al.*, 2020).

Unilateral ureteral-obstruction in mice results in the upregulation of H3-3B, a CKD candidate, in the kidneys (Shindo *et al.*, 2018). In addition, a knockdown experiment of histone cell cycle regulation defective homolog A (HIRA) in a normal rat kidney cell (NRK-52) causes the decreased H3-B3 expression and increased fibrogenesis (Shindo *et al.*, 2018). Furthermore, in patients with IgA nephropathy, H3-3B immune-stains positively correlate with kidney fibrosis (Shindo *et al.*, 2018). These results support a therapeutic candidacy of H3-3B for renal diseases including CKD.

Genetic mutation of PYGL, a candidate for both CKD and MI, in human prevents effective glycogen breakdown in the liver leading to glycogen storage diseases (Zhan *et al.*, 2021). While no clinical implications for CKD or MI or other renal/cardiovascular diseases in the patients of these conditions are recorded (NCT02385162), our expression analysis (Fig. 5) suggests that the liver is a potential therapeutic target for both CKD and MI. Hence, PYGL could serve as a therapeutic target for CKD, MI and/or other renal/cardiovascular diseases.

UBE2D1, a CKD candidate, is inferred as a potential biomarker for diabetes-related sepsis by a machine-learning pipeline using public databases (Wang *et al.*, 2022). Diabetes is a known risk factor for CKD (Shahbazian and Rezaii, 2013), hence, further supporting the candidacy of UBE2D1 for a CKD target as deduced herein.

These retrospective and independent validation results in Table 1 further support the therapeutic possibilities of the candidates reported in this study for CKD and/or MI and/or other renal and/or cardiovascular diseases. At the same time, they also strengthen the possibility that the predicated-targets without the retrospective evidence (i.e. those with blank in both the Publication and ClinicalTrials.gov cells) would represent new disease-omics features for treating and/or detecting CKD and/or MI.

## 4 Discussion

In this article, we applied a multi-modal soft-clustering method to the multiple disease-omics datasets and uncovered latent molecular similarities across 6955 human diseases (Figs 1–4). By exploiting these molecular similarities, we identified 69 targets that could be therapeutically repurposed for CKD and/or MI treatments (Table 1). The comprehensive omics analyses, in combination with an edit-distance-based classifier, found their underlying therapeutic mechanisms (Figs 5, 6, 8 and 9).

Importantly, we found the evidence retrospectively supporting the predicted therapeutic utility of the 17 targets in the pre-clinical and clinical data space (Table 1).

Recently, other soft-clustering methods for multi-modal data are reported (Yan *et al.*, 2021; Zhang *et al.*, 2022). While their utility or effectiveness with multi-modal biomedical data remains unknown, they may provide an additional framework to the analysis of the multi-modal disease-omics data studied in this article.

Our primary motivation of this study is to identify repurposable disease-omics molecular features that could be repurposed from a disease to another disease to its treatment and/or detection on the basis of multi-modal disease–disease similarities that are underrecognized in the conventional disease classification. Toward this goal, we used the soft-clustering of human diseases by multi-modal generative topic modeling to detect subtle differences in the multi-modal features of the diseases (Fig. 1). As a result, we were able to develop an algorithm that exhibits mostly AUC > 0.8 for predicting the missing modality features of 6955 human diseases (Fig. 2A). In this study, we tested this method with three modalities, Ae, Bm, Gv, for 6955 diseases (Fig. 2A), and four modalities, Ae, Bm, Gv, Mb, for 158 diseases (Fig. 2B). The result shows both sets result in virtually equivalent performance, suggesting the scalability of the method with additional modalities.

This method identified molecular features shared by CKD/MI and non-renal/non-cardiovascular diseases such as neoplasia and mental/neurological disorders (Fig. 4), indicating a latent underlying mechanism shared among these diseases. The neoplasia can be regarded as a partial cellular reprogramming, as it is accompanied by the aberrant activations of large number of genes (Buganim *et al.*, 2012; Suvà *et al.*, 2013; Ward and Thompson, 2012; Xing *et al.*, 2020). This phenomenon could be reflected in the molecular similarities between the renal/cardiovascular diseases (e.g. CKD and MI) and the neoplasia. It is also recently reported that MI accelerates breast cancer via innate immune reprogramming (Koelwyn *et al.*, 2020). This clinical observation might be a consequence of their molecular and mechanistic similarities as predicted in this study. Moreover, various clinical observations also suggest that CKD and cancer are mutual risk-factors, but without any clear molecular mechanisms (Wong *et al.*, 2016). Hence, it is possible that the predicted molecular mechanisms/pathways shared by CKD and neoplasia reported in this article may be an underlying molecular mechanism of these clinical observations.

Virtually all peripheral organs such as the liver, the kidney, the heart, etc. are under the control of neural inputs and these organs feedback their physiological information to the neural organ such as the brain (Imai *et al.*, 2008; Underwood and Altounian, 2021). Hence, such inter-organ neural feed-forward and feed-back loops could be reflected in the similar molecular features and underlying mechanisms of the renal/cardiovascular diseases (e.g. CKD and MI) and the mental/neurological disorders as predicted by the method reported herein. In support of this possibility, many mental disorders are prevalent in CKD patients (Simoes *et al.*, 2019). Furthermore, myocardial infarction is often followed by deteriorated mental health conditions (De Hert *et al.*, 2018; Lloyd, 1987). Despite such clinical evidence, no concrete molecular mechanisms explaining these clinical observations remain unknown. Thus, the common molecular mechanisms and/or pathways described in this study could be the ones.

This study identified 69 molecules that could be targeted for the treatment and/or detection of CKD and/or MI treatments on the basis of the similarities of CKD and/or MI to neoplasia and/or mental/neurological disorders (Table 1). Their expression patterns and KEGG/GO analyses indicate they are enriched in the brain and the metabolic organs such as the liver and their physiological functions (Figs 5 and 6). These results are coherent with the molecular similarities between the CKD/MI and mental/neurological disorders described in this article. They are also consistent with the fact that many of the renal/cardiovascular diseases including CKD and MI are broadly regarded as metabolic and life-style diseases (Sharifi-Rad *et al.*, 2020; Thomas *et al.*, 2011).

The edit-distance-based classifier shows two types of therapeutic mechanisms by which these 69 candidates could elicit their effects in the treatments of CKD, MI and/or other renal-/cardiovascular-diseases. Those that function via the pathways that are also targeted by the drugs approved for the corresponding diseases (i.e. CKD, MI), and the others that function independently of them (Fig. 8). The independent pathways may be a part of the previously unknown molecular mechanisms underlying the corresponding disease(s). In this case, their therapeutic-targeting could lead to the development of ‘first-in-class’ drugs for the corresponding diseases. In contrast, those within the already-targeted pathways are activated or inhibited by the existing drugs. Hence, they could be further developed by adding new indications for the diseases that are described as molecularly similar in this article.

The edit-distance-based classifier is also applied to evaluate putative SEs that could accompany the therapeutic targeting of these candidate molecules (Fig. 9). The result shows the four (AK2, ASL, PILRA, PYGL) are less harmful targets. This analysis provides useful information for selecting out those that are likely less toxic, prior to spending labor-, time- and cost-intensive pre-clinical and clinical studies during the therapeutic development.

The likeliness of the repurposability of the predicted CKD and MI targets is further strengthened by the retrospective finding of the therapeutic implications of the 17 targets in the pre-clinical and clinical-trials data space, despite their absence in the training datasets (Table 1). In addition to these molecular targets with the retrospective evidence, we also found those without any retrospective evidence and they may represent new molecular features that could therapeutically be developed to treat and/or detect CKD and/or MI.

In this study, we introduced a multi-modal generative topic modeling approach to find repurposable molecular targets and their use to unveil latent disease–disease similarities, and the characterization of their suitability for therapeutic development by the edit-distance-based method. While the results show many promises, there are some limitations that must be noted:

1. The multi-modal generative topic modeling computes the disease similarities according to the known multi-modal features of one or more of the human diseases. Hence, the features unlinked to any of the diseases are excluded from the outputs.
2. A caution must be paid to the interpretation of the cell-type expression (Fig. 5B) and the enrichment results (Fig. 5D). This is due to the apparent biases in the cell-type representations in the currently available human single-cell transcriptome databases,
3. All paths in the edit-distance classifier are generated with a target as the starting node. Hence, if the target is the most downstream component, it cannot be analyzed by this approach.
4. The edit-distance classifier reported herein is a mechanism-based predictor. Hence, any ‘off-target’ effects are out of scope.
5. The approach introduced here does not consider ‘druggability’ of the identified molecules, which are also important factors to determine whether the targets are repurposable (Owens, 2007).

Despite such limitations, the retrospective validation (Table 1), together with the high AUC scores obtained by the cross-validation (Figs 2 and 3) and the modality scalability (Fig. 2), demonstrates the effectiveness of the method in uncovering latent disease–disease similarities and therapeutic repurposing possibilities across diverse diseases and modalities. Hence, the method is expected to be effective, not only for CKD or MI, but also for other types of diseases and with different and/or additional combinations of disease feature modalities.

## Supplementary Material

vbad047_Supplementary_Data

## Data Availability

The code and curated data reported in this article are available at: https://github.com/skozawa170301ktx/MultiModalDiseaseModeling.
