# Supplementary material for: Latent disease similarities and therapeutic repurposing possibilities uncovered by multi-modal generative topic modeling of human diseases
Source: Bioinform Adv. 2023 Apr 12;3(1):vbad047. doi: 10.1093/bioadv/vbad047 (PMC10133403; doi:10.1093/bioadv/vbad047)
Supplement: vbad047_Supplementary_Data [file vbad047_supplementary_data.zip › BIOADV-2022-169.R2_SupplementaryInformation_tns.pdf]

Supplementary Information

Latent disease similarities and therapeutic repurposing possibilities uncovered by multi-modal generative topic modeling of human diseases

Satoshi Kozawa[1,2,3], Hirona Yokoyama[1,2,4], Kyoji Urayama[1,2,3], Kengo Tejima[1,2,3], Hotaka Doi[1,2,4], Shunki Takagi[1,2], Thomas N. Sato[1,2,3,4 ¶]

[1]Karydo TherapeutiX, Inc., Kyoto, Japan; [2]The Thomas N. Sato BioMEC-X Laboratories, Advanced Telecommunications Research Institute International (ATR), Kyoto, Japan; [3]ERATO Sato-Live Bio-Forecasting Project, Kyoto, Japan; [4]V-iCliniX Laboratory, Nara Medical University, Nara, Japan

[¶]Corresponding Author

Thomas N. Sato, Ph.D.

[1]Karydo TherapeutiX, Inc., Kyoto, Japan

2-2-2 Hikaridai, Seika-cho, Soraku-gun, Kyoto 619-0288 Japan

TEL: +81-3-6671-9094

Email: island1005@gmail.com

Figure S1

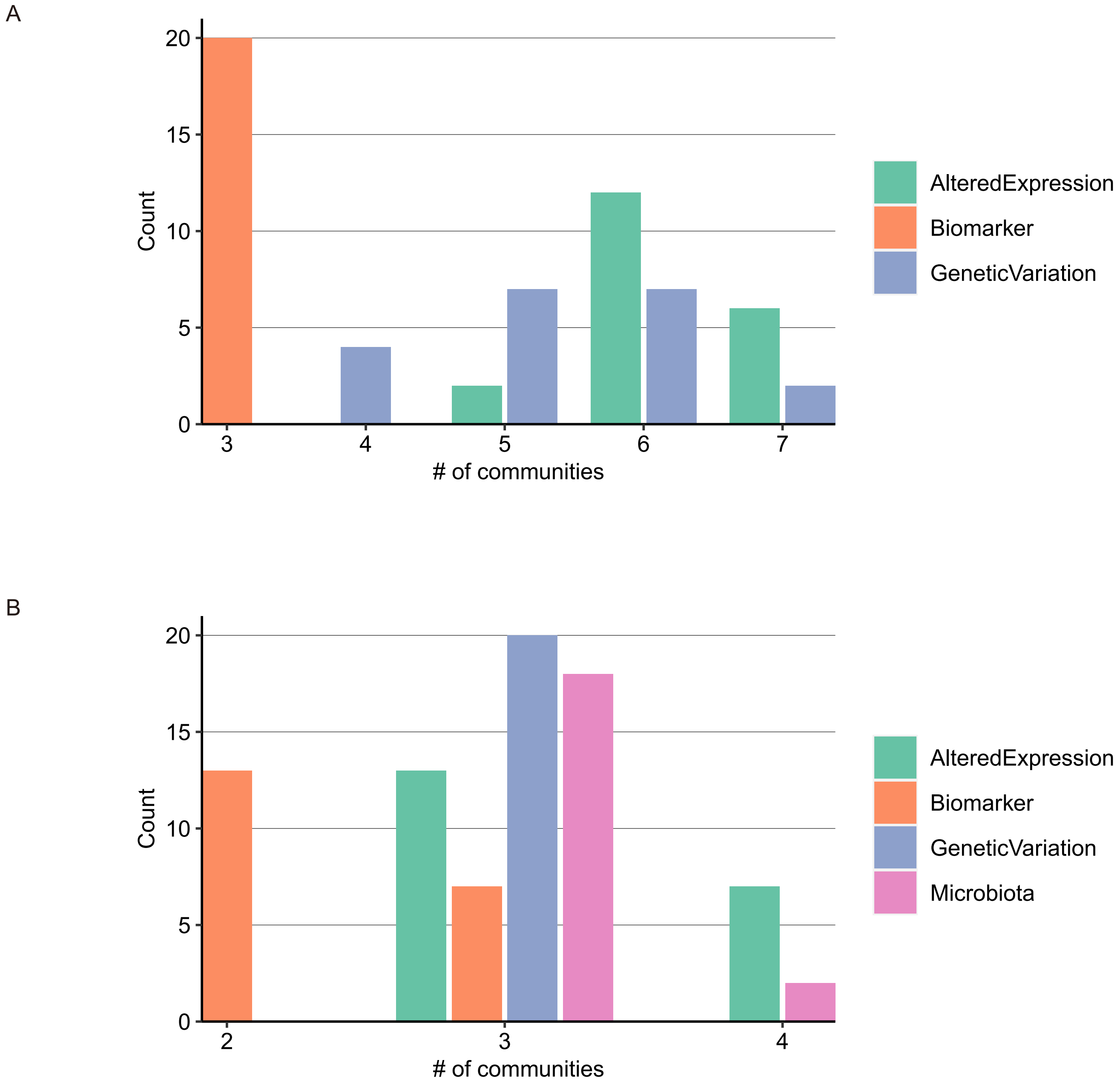

Figure S1. The community numbers, Related to Figure 1.
(A) The numbers of communities found by the community detection method for the Ae/Bm/Gv datasets encompassing 6,955 diseases are shown as bar graph for each disease-omics modality.
(B) The numbers of communities found by the community detection method for the Ae/Bm/Gv/Mb datasets encompassing 158 diseases are shown as bar graph for each disease-omics modality.

Table S1. The raw data of Figure 2A

Database: disease modality, key_c: UMLS ID of the corresponding disease – otherwise disease name, Disease_ID: disease number in the figure, Disease: disease name in the database (DisGeNET v. 7.0), AUC: AUC score.

Table S2. The raw data of Figure 2B

Database: disease modality, key_c: UMLS ID of the corresponding disease – otherwise disease name, Disease_ID: disease number in the figure, Disease: disease name in the database (Disbiome for Mb, DisGeNET v. 7.0 for the others), AUC: AUC score.

Table S3. The raw data of the AUC scores in Figure 3

element_name: disease-omics modality name, Disease: disease name, auc: AUC score.

Table S4. The list of the predicted Ae features for CKD, Related to Figure 3.

The list of the predicted features (component) for Ae (AlteredExpression) modality for the chronic kidney disease (Disease). The count number (Count) and the frequency (Freq) in the prediction outputs are shown for each feature (component). The presence (1) and absence (0) of the predicted feature (component) in the training data are indicated in the "Obs" column.

Table S5. The list of the predicted Bm features for CKD, Related to Figure 3.

The list of the predicted features (component) for Bm (Biomarker) modality for the chronic kidney disease (Disease). The count number (Count) and the frequency (Freq) in the prediction outputs are shown for each feature (component). The presence (1) and absence (0) of the predicted feature (component) in the training data are indicated in the "Obs" column.

Table S6. The list of the predicted Gv features for CKD, Related to Figure 3.

The list of the predicted features (component) for Gv (GeneticVariation) modality for the chronic kidney disease (Disease). The count number (Count) and the frequency (Freq) in the prediction outputs

are shown for each feature (component). The presence (1) and absence (0) of the predicted feature (component) in the training data are indicated in the “Obs” column.

Table S7. The list of the predicted Ae features for MI, Related to Figure 3.

The list of the predicted features (component) for Ae (AlteredExpression) modality for the myocardial infarction (Disease). The count number (Count) and the frequency (Freq) in the prediction outputs are shown for each feature (component). The presence (1) and absence (0) of the predicted feature (component) in the training data are indicated in the “Obs” column.

Table S8. The list of the predicted Bm features for MI, Related to Figure 3.

The list of the predicted features (component) for Bm (Biomarker) modality for the myocardial infarction (Disease). The count number (Count) and the frequency (Freq) in the prediction outputs are shown for each feature (component). The presence (1) and absence (0) of the predicted feature (component) in the training data are indicated in the “Obs” column.

Table S9. The list of the predicted Gv features for MI, Related to Figure 3.

The list of the predicted features (component) for Gv (GeneticVariation) modality for the myocardial infarction (Disease). The count number (Count) and the frequency (Freq) in the prediction outputs are shown for each feature (component). The presence (1) and absence (0) of the predicted feature (component) in the training data are indicated in the “Obs” column.

Table S10. The Youden’s index for each modality feature, Related to Figure 3.

Database: modality, Disease: disease name.

Table S11. The list of the predicted Ae features above the corresponding Youden’s index for CKD following the removal of those in the training data, Related to Figure 3.

Database: modality, key_c: UMLS ID of the corresponding disease (i.e., the chronic kidney disease), Disease: the chronic kidney disease, component: the name of the predicted feature, keg component id:

KEGG ID number for the predicted feature (component), obs: 0 as they are absent in the training data, sample count: count number in the output, freq: frequency in the output, roc_cutoff: the threshold cut-off value based on the Youden's index, DisGeNET Database: the modality in the DisGeNET database (i.e., the training data) where the corresponding feature (component) appears, DisGeNET Disease: the disease in the DisGeNET database (i.e., the training data) where the corresponding feature (component) appears.

Table S12. The list of the predicted Bm features above the corresponding Youden's index for CKD following the removal of those in the training data, Related to Figure 3.

Database: modality, key_c: UMLS ID of the corresponding disease (i.e., the chronic kidney disease), Disease: the chronic kidney disease, component: the name of the predicted feature, keg component id: KEGG ID number for the predicted feature (component), obs: 0 as they are absent in the training data, sample count: count number in the output, freq: frequency in the output, roc_cutoff: the threshold cut-off value based on the Youden's index, DisGeNET Database: the modality in the DisGeNET database (i.e., the training data) where the corresponding feature (component) appears, DisGeNET Disease: the disease in the DisGeNET database (i.e., the training data) where the corresponding feature (component) appears.

Table S13. The list of the predicted Gv features above the corresponding Youden's index for CKD following the removal of those in the training data, Related to Figure 3.

Database: modality, key_c: UMLS ID of the corresponding disease (i.e., the chronic kidney disease), Disease: the chronic kidney disease, component: the name of the predicted feature, keg component id: KEGG ID number for the predicted feature (component), obs: 0 as they are absent in the training data, sample count: count number in the output, freq: frequency in the output, roc_cutoff: the threshold cut-off value based on the Youden's index, DisGeNET Database: the modality in the DisGeNET database (i.e., the training data) where the corresponding feature (component) appears, DisGeNET Disease: the disease in the DisGeNET database (i.e., the training data) where the corresponding feature (component) appears.

Table S14. The list of the predicted Ae features above the corresponding Youden's index for MI following the removal of those in the training data, Related to Figure 3.

Database: modality, key_c: UMLS ID of the corresponding disease (i.e., the myocardial infarction), Disease: the myocardial infarction, component: the name of the predicted feature, keg component id: KEGG ID number for the predicted feature (component), obs: 0 as they are absent in the training data, sample count: count number in the output, freq: frequency in the output, roc_cutoff: the threshold cut-off value based on the Youden's index, DisGeNET Database: the modality in the DisGeNET database (i.e., the training data) where the corresponding feature (component) appears, DisGeNET Disease: the disease in the DisGeNET database (i.e., the training data) where the corresponding feature (component) appears.

Table S15. The list of the predicted Bm features above the corresponding Youden's index for MI following the removal of those in the training data, Related to Figure 3.

Database: modality, key_c: UMLS ID of the corresponding disease (i.e., the myocardial infarction), Disease: the myocardial infarction, component: the name of the predicted feature, keg component id: KEGG ID number for the predicted feature (component), obs: 0 as they are absent in the training data, sample count: count number in the output, freq: frequency in the output, roc_cutoff: the threshold cut-off value based on the Youden's index, DisGeNET Database: the modality in the DisGeNET database (i.e., the training data) where the corresponding feature (component) appears, DisGeNET Disease: the disease in the DisGeNET database (i.e., the training data) where the corresponding feature (component) appears.

Table S16. The list of the predicted Gv features above the corresponding Youden's index for MI following the removal of those in the training data, Related to Figure 3.

Database: modality, key_c: UMLS ID of the corresponding disease (i.e., the myocardial infarction), Disease: the myocardial infarction, component: the name of the predicted feature, keg component id: KEGG ID number for the predicted feature (component), obs: 0 as they are absent in the training data,

sample count: count number in the output, freq: frequency in the output, roc_cutoff: the threshold cut-off value based on the Youden's index, DisGeNET Database: the modality in the DisGeNET database (i.e., the training data) where the corresponding feature (component) appears, DisGeNET Disease: the disease in the DisGeNET database (i.e., the training data) where the corresponding feature (component) appears.

Table S17. The final list of the 30 predicted targets proteins for CKD, Related to Figure 3.
They are indicated by the gene/protein symbol (Symbol).

Table S18. The final list of the 57 predicted targets proteins for MI, Related to Figure 3.
They are indicated by the gene/protein symbol (Symbol).

Table S19. The raw data of Figure 4.
Each disease is indicated by UMLS ID (key_c) when available (otherwise, it is the same as the disease name). Each is indicated for CKD or MI (CKD_MI) and how many of the predicted genes for the corresponding disease appears in the training data for the corresponding disease (n_genes). The "n-genes" corresponds to "Count" in Figure 4.

Table S20. The raw data of Figure 5A.
The target is indicated as gene symbol (Symbol). The organs (Tissue) where the corresponding target is expressed and its expression level (RNAExp) are shown.

Table S21. The raw data for Figure 5B.
The target is indicated as gene symbol (Symbol). The cell-types (SCT) where the corresponding target is expressed and its expression level (RNAExp) are shown.

Table S22. The raw data for Figure 5C.

The organ name (Tissue) and its p-value (pvalue), adjusted p-value (padj), and q-value (qvalue) are shown.

Table S23. The raw data for Figure 5D.

The cell-type name (SCT) and its p-value (pvalue), adjusted p-value (padj), and q-value (qvalue) are shown.

Table S24. The raw data for Figure 6.

Those with padj<0.05 are listed.

Table S25. The KEGG pathway enrichment result for the CKD targets.

Those with p.adjust<0.5 are listed.

Table S26. The GO BP enrichment result for the CKD targets.

Those with p.adjust<0.5 are listed.

Table S27. The GO CC enrichment result for the CKD targets.

Those with p.adjust<0.5 are listed.

Table S28. The GO MF enrichment result for the CKD targets.

Those with p.adjust<0.5 are listed.

Table S29. The holdout validation of the therapeutic-indication (TI) prediction by the edit-distance based classifier (Method A), Related to Figure 7.

Table S30. The holdout validation of the side-effect (SE) prediction by the edit-distance based classifier (Method B), Related to Figure 7.

Table S31. The raw data (CKD, Method A) of Figure 8.

TI: the target disease (CKD), KEGG component_id: KEGG ID of the corresponding target, description: the name of the target, symbol: the symbol of the target, prob: probability of the prediction of the corresponding target, pathway: whether the target is in the same KEGG pathway as the clinically approved therapeutic target in the training data (1: in the same pathway, 0: in a different pathway)

Table S32. The raw data (CKD, Method B) of Figure 8.

TI: the target disease (CKD), KEGG component_id: KEGG ID of the corresponding target, description: the name of the target, symbol: the symbol of the target, prob: probability of the prediction of the corresponding target, pathway: whether the target is in the same KEGG pathway as the clinically approved therapeutic target in the training data (1: in the same pathway, 0: in a different pathway)

Table S33. The raw data (MI, Method A) of Figure 8.

TI: the target disease (MI), KEGG component_id: KEGG ID of the corresponding target, description: the name of the target, symbol: the symbol of the target, prob: probability of the prediction of the corresponding target, pathway: whether the target is in the same KEGG pathway as the clinically approved therapeutic target in the training data (1: in the same pathway, 0: in a different pathway)

Table S34. The raw data (MI, Method B) of Figure 8.

TI: the target disease (MI), KEGG component_id: KEGG ID of the corresponding target, description: the name of the target, symbol: the symbol of the target, prob: probability of the prediction of the corresponding target, pathway: whether the target is in the same KEGG pathway as the clinically approved therapeutic target in the training data (1: in the same pathway, 0: in a different pathway)

Table S35. The holdout validation of the side-effect (SE) prediction by the edit-distance based classifier (Methods A and B). The raw data of Figure 9. Each SE_ID in Figure 9 is fully-described in the table.

Table S36. The raw data of Table 1.

The KEGG component IDs of the targets, the KEGG pathway IDs to which the targets belong to, GO IDs of the targets are also added to this table.
