# Supplementary material for: Latent disease similarities and therapeutic repurposing possibilities uncovered by multi-modal generative topic modeling of human diseases
Source: Bioinform Adv. 2023 Apr 12;3(1):vbad047. doi: 10.1093/bioadv/vbad047 (PMC10133403; doi:10.1093/bioadv/vbad047)
Supplement: vbad047_Supplementary_Data [file vbad047_supplementary_data.zip › FigureS1.pdf]

Figure S1

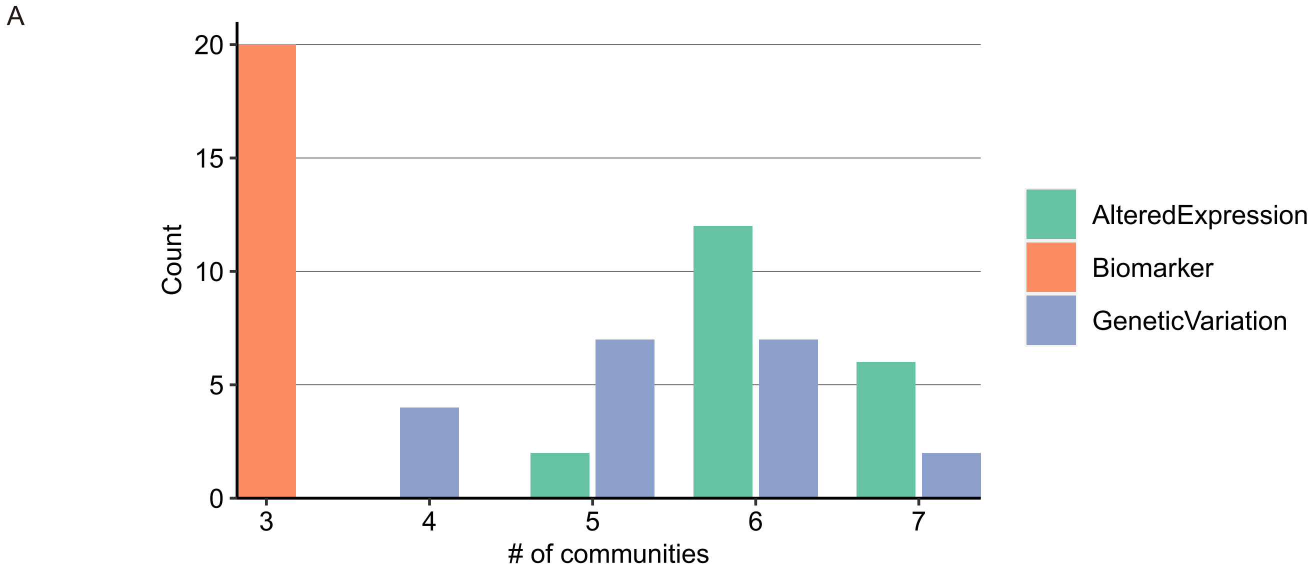

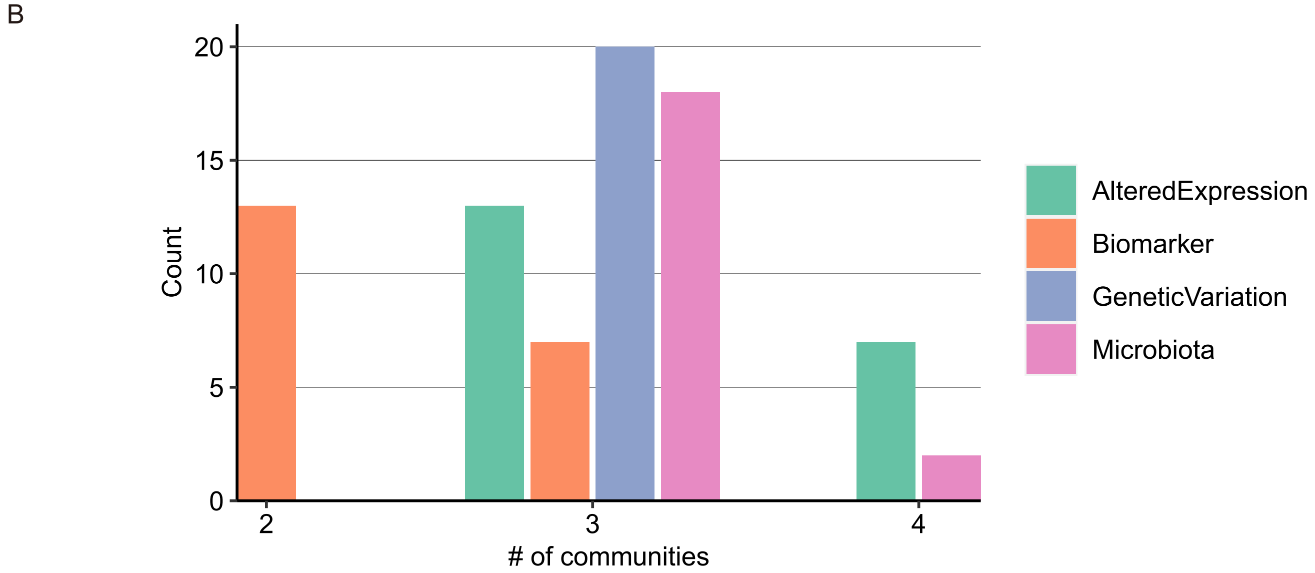

Figure S1. The community numbers, Related to Figure 1.
(A) The numbers of communities found by the community detection method for the Ae/Bm/Gv datasets encompassing 6,955 diseases are shown as bar graph for each disease-omics modality.
(B) The numbers of communities found by the community detection method for the Ae/Bm/Gv/Mb datasets encompassing 158 diseases are shown as bar graph for each disease-omics modality.
